# Fast approximate inference for variable selection in Dirichlet process mixtures, with an application to pan-cancer proteomics

**DOI:** 10.1515/sagmb-2018-0065

**Published:** 2019-12-12

**Authors:** Oliver M. Crook, Laurent Gatto, Paul D.W. Kirk

**Affiliations:** 1 Department of Applied Mathematics and Theoretical Physics, University of Cambridge, Cambridge, UK; 2 Department of Biochemistry, Cambridge Centre for Proteomics, University of Cambridge, Cambridge, UK; 3 MRC Biostatistics Unit, School of Clinical Medicine, University of Cambridge, Cambridge, UK; 4 UCLouvain, de Duve Institute, Brussels, Belgium; 5 University of Cambridge, Cambridge Institute of Therapeutic Immunology & Infectious Disease (CITIID), Cambridge Biomedical Campus Cambridge, United Kingdom of Great Britain and Northern Ireland

**Keywords:** Bayesian clustering, cancer proteomics, variable selection

## Abstract

The Dirichlet Process (DP) mixture model has become a popular choice for model-based clustering, largely because it allows the number of clusters to be inferred. The sequential updating and greedy search (SUGS) algorithm (Wang & Dunson, 2011) was proposed as a fast method for performing approximate Bayesian inference in DP mixture models, by posing clustering as a Bayesian model selection (BMS) problem and avoiding the use of computationally costly Markov chain Monte Carlo methods. Here we consider how this approach may be extended to permit variable selection for clustering, and also demonstrate the benefits of Bayesian model averaging (BMA) in place of BMS. Through an array of simulation examples and well-studied examples from cancer transcriptomics, we show that our method performs competitively with the current state-of-the-art, while also offering computational benefits. We apply our approach to reverse-phase protein array (RPPA) data from The Cancer Genome Atlas (TCGA) in order to perform a pan-cancer proteomic characterisation of 5157 tumour samples. We have implemented our approach, together with the original SUGS algorithm, in an open-source R package named sugsvarsel, which accelerates analysis by performing intensive computations in C++ and provides automated parallel processing. The R package is freely available from: https://github.com/ococrook/sugsvarsel

## 1 Introduction

Bayesian nonparametric methods have become commonplace in the statistics and machine learning literature due to their flexibility and wide applicability. For model-based clustering, Dirichlet process (Ferguson 1973; 1974) mixture models have become particularly popular (Antoniak, 1974; Lo, 1984; Escobar, 1994; Escobar & West, 1995; Blei & Jordan, 2006), partly because they allow the number of clusters supported by the data to be inferred. By introducing latent selection indicators, these models can be extended to perform variable selection for clustering (Kim, Tadesse & Vannucci, 2006), which is particularly relevant in high-dimensional settings (Law, Figueiredo & Jain, 2004; Constantinopoulos, Titsias & Likas, 2006). There are now several approaches for modelbased clustering and variable selection (see Fop & Murphy, 2018, for a recent review), but current Markov chain Monte Carlo (MCMC) algorithms for Bayesian inference in Dirichlet process (DP) mixture models (e.g. Neal, 2000; Jain & Neal, 2004) are computationally costly, and often infeasible for large datasets.

A number of algorithms have been proposed for fast approximate inference in DP and related mixture models, which make possible the analysis of datasets with large numbers of observations. In the present paper, we focus on the sequential updating and greedy search (SUGS) algorithm (Wang & Dunson, 2011; Zhang et al., 2014), which we describe in more detail in Section 2.2. However, there are many other approximate inference procedures, a (non-exhaustive, but representative) selection of which we now briefly describe. Variational Bayes (VB) approaches for approximate inference in mixture models have a long history (Attias 1999; 2000), and were extended to DP mixture models by Blei and Jordan (2006). Despite well-known limitations in terms of generally underestimating the variance of the posterior, variational techniques have enabled (approximate) Bayesian inference to be applied to a large class of models and “big data” settings, and are now a mainstay of modern computational Bayesian statistics (Blei, Kucukelbir & McAuliffe, 2016). We note that SUGS was previously shown by Wang and Dunson (2011) to be 10 times faster than VB (largely due to the authors finding that VB required a computationally costly initialisation step in order to provide good results), while performing comparably to VB in terms of model fit. Daumé III (2007) provided an alternative approximate inference strategy that uses fast search algorithms to seek the maximum *a posteriori* (MAP) allocation of observations to clusters, and demonstrated that these techniques permit clustering of very large datasets. The results obtained depend upon the order in which observations are considered, and hence Daumé III (2007) considered a number of ordering strategies. *Bayesian hierarchical clustering* (Heller & Ghahramani, 2005; Savage et al., 2009; Cooke et al., 2011; Darkins et al., 2013) is another method for performing approximate inference for a DP mixture model that also identifies a single optimal clustering structure, but does so using an agglomerative hierarchical clustering approach that determines which clusters to merge at each step on the basis of computed marginal likelihoods. In contrast, by revisiting the widely used *k*-means algorithm from a Bayesian nonparametric viewpoint, Kulis and Jordan (2012) proposed a novel hard clustering algorithm called *DP-means*, which was subsequently generalised beyond the Gaussian mixtures case (Jiang, Kulis & Jordan, 2012) and was also adapted to cluster large sequencing datasets (Jiang et al., 2016). The MAP-DP approach of Raykov, Boukouvalas, and Little (2016a) is an approximate maximum *a posteriori* inference algorithm for DP mixtures, which has also been proposed as a principled alternative to *k*-means (Raykov et al., 2016b), but which – in contrast to DP-means – inherits the “rich get richer” property of the DP mixture model, and allows standard model selection and model fit diagnostics to be used (Raykov, Boukouvalas & Little, 2016a). Despite the advances provided by the above methods in terms of reduced computational cost and scalability to large datasets, we note that without variable selection all of these approaches may be ill-suited in high-dimensional settings.

In the spirit of the original SUGS algorithm, here we pose clustering and variable selection as a Bayesian model selection (BMS) problem. We consider variable selection for clustering in terms of partitioning variables into those which are relevant and those which are irrelevant for defining the clustering structure, and thereby pose the problem as one of using BMS to select both a partition of the variables and a partition of the observations. We moreover consider the benefits of performing Bayesian model averaging (BMA) (Madigan & Raftery, 1994; Hoeting et al., 1999) for summarising the SUGS output. For ease of exposition, we focus on the case of DP Gaussian mixtures, but note that all of our methods extend straightforwardly to other distributions for which conjugate priors may be chosen.

We consider a range of simulation settings and well-studied examples from cancer transcriptomics to show that our methods perform competitively with the current state-of-the-art. Having established the utility of our approach, we consider an application to reverse-phase protein arrays (RPPA) datasets in order to characterise the pan-cancer functional proteome. Such datasets have the potential to provide a deeper understanding of the biomolecular processes at work in cancer cells, and have previously been shown to offer additional insights beyond what may be captured by genomics or transcriptomics datasets (Akbani et al., 2014). Here we consider RPPA data for 5157 tumour samples obtained from The Cancer Genome Atlas (TCGA).

Section 2 recaps DP mixture models and the SUGS algorithm, then describes our extensions to SUGS including variable selection and BMA. Section 3 evaluates our method on simulated datasets and compares it with other approaches to clustering and variable selection. We then apply our method to a large proteomics dataset, highlighting its applicability. In the final section, we make some concluding remarks and discuss limitations and extensions. Our methods are implemented in an R package: https://github.com/ococrook/sugsvarsel.

## 2 Methods

### 2.1 Dirichlet process mixtures

We provide a very brief recap of DP mixture models, mainly to introduce notation, and refer to the overview provided in Section 3 of Teh et al. (2006) for further details. Let *G* ~ *DP*(*βP*_0_) where *β* > 0 is the DP concentration parameter, *P*_0_ is the base probability measure, and *G* is a random probability measure. We consider a Pólya urn scheme in which we have independent and identically distributed (i.i.d.) random variables *θ*_1_, *θ*_2_, … distributed according to *G*. Computing the sequential conditional distributions of *θ_i_* given *θ*_1_, …, *θ_i−1_*, upon marginalising out the random *G*, we obtain (Blackwell & MacQueen, 1973):

(1) $$\theta_{i}|\theta_{1},\ldots ,\theta_{i-1}∼\frac{\beta }{\beta +i-1}P_{0}+\frac{1}{\beta +i-1}\sum_{i-1}^{l=1}\delta_{\theta_{l^{\prime }}}i=1,\ldots ,n,$$

where *δ_θ_* is a probability measure with mass concentrated at *θ*. It is clear from this equation that for any *r* = 1, 2, …, i − 1, the probability that *θ_i_* is equal to *θ_r_* is given by $\sum_{l=1}^{i=1}𝕀\left(\theta_{l}=\theta_{r}\right)/(\beta +i-1)$, where 𝕀(*X*) = 1 if *X* is true and 𝕀(*X*) = 0 otherwise. Thus *θ_i_* has non-zero probability to be equal to one of the previous draws, and it is this clustering property that makes the DP a suitable prior for mixture models.

The DP mixture model is obtained by introducing an additional parametric probability distribution, *F*. More precisely, let observations *x_i_* be modelled according to the following hierarchical model:

(2) $$\begin{array}{l} G∼DP(\beta P_{0}),\\ \theta_{i}|G∼G,\\ x_{i}|\theta_{i}∼F(\theta_{i}), \end{array}$$

where *F* denotes the conditional distribution of the observation *x_i_* given *θ_i_*. For example, when *F* is chosen to be a Gaussian random variable we arrive at the DP Gaussian mixture model (also referred to as the infinite Gaussian mixture model; Rasmussen, 2000).

When performing inference for such models, it is common to introduce a set of latent variables (cluster labels) *z*_1_, …, *z_n_* associated with the observations, such that *z_i_* is the cluster label for observation *x_i_*. From the above specification of the DP mixture model, it follows that the conditional prior distribution of *z_i_* given *z*_−*i*_ = (*z*_1_, …, *z*_*i*−1_) is categorical with:

(3) $$\pi_{lk}:=P\left(z_{i}=k|z_{-i},\beta \right)=\{\begin{array}{ll} \frac{n_{k}}{\beta +1-1}, & \text{for }k=1,..,K-1\\ \frac{\beta }{\beta +1-1}, & \text{for}\;k=K, \end{array}$$

where *β* > 0 is the DP concentration parameter, *n_k_* := $\sum_{l=1}^{i=1}𝕀\left(z_{l}=k\right)$ is the number of previous observations allocated to cluster *k*, and *K* = max{*z*_−*i*_} + 1. Larger values of *β* encourage observations to be allocated to new clusters, hence *β* plays a role in controlling the number of clusters.

Inference for DP mixture models can performed using computationally intensive MCMC methods (Neal, 2000; Jain & Neal, 2004). However, as we discuss below, here we are interested in the SUGS algorithm for approximate inference, proposed by Wang and Dunson (2011).

### 2.2 Sequential updating and greedy search (SUGS)

SUGS is a sequential approach for allocating observations to clusters, which (greedily) allocates the *i*-th observation to a cluster, given the allocations of the previous *i* − 1 observations. Suppose that observations *x*_−*i*_ = (*x*_1_, …, *x*_*i*−1_) have previously been allocated to clusters. As described in Wang and Dunson (2011), the posterior probability of allocating observation *i* to cluster *k* according to the DP mixture model formulation above is given by:

(4) $$P\left(z_{i}=k|x_{i},x_{-i},z_{-i},\beta \right)=\frac{\pi_{ik}L_{ik}\left(x_{i}\right)}{\sum_{l=1}^{K}\pi_{ik}L_{il}\left(x_{i}\right)},$$

where *π_ik_* is defined as in Equation (3), and

(5) $$L_{ik}=\int f\left(x_{i}|\theta_{k}\right)p\left(\theta_{k}|x_{-i},z_{-i}\right)d\theta_{k}$$

is the conditional marginal likelihood associated with *x_i_* given allocation to cluster *k* and the cluster allocations for observations 1, …, *i*−1, with *f*(*x_i_*|*θ_k_*) denoting the likelihood associated with *x_i_* as a function of *θ_k_*. If *k* is a cluster to which previous observations have already been allocated, then *p*(*θ_k_*|*x*_−*i*_,*z*_−*i*_) is the posterior distribution of *θ_*k*_* given the observations previously allocated to cluster *k*; i.e. $p\left(\theta_{k}|x_{-i},z_{-i}\right)\propto p_{0}\left(\theta_{k}\right)\prod_{j:z_{j}=k,1\leq j\leq i-1}f\left(x_{j}|\theta_{k}\right)$, where *p*_0_(*θ_k_*) is the prior on the cluster-specific parameters, *θ_k_*. For a new cluster, i.e. for *k* = *K*, we have *p*(*θ_k_*|*x*_−*i*_,*z*_−*i*_) = *p*_0_(*θ_k_*). If *P*_0_ is taken to be conjugate for the likelihood *f*, then the posterior and conditional marginal likelihood are available analytically.

Assuming that the concentration parameter *β* is given and that conjugate priors are taken, the above suggests a computationally efficient deterministic clustering algorithm (the SUGS algorithm). That is, *z*_1_ is initialised as *z*_1_ = 1, and then subsequent observations are sequentially allocated to clusters by setting *z_i_* = arg max_*k*∈{1,…,*K*}_ *P*(*z_i_* = 𝑘|*x_i_*,*x*_−*i*_,*z*_−*i*_, *β*), where we recall that *K* = max{*z*_−*i*_} + 1 may change after each sequential allocation.

#### 2.2.1 Dealing with unknown *β*

The DP concentration parameter *β* directly influences the number of clusters, thus we treat this as a random variable to be inferred, in the same way as in Wang and Dunson (2011). In particular, let $\hat{\beta }=\left({\hat{\beta }}_{1},\ldots ,{\hat{\beta }}_{L}\right)$ be a discrete grid of permissible values for *β* with a large range, and then define the prior for *β* to be discrete with the following form:

(6) $$p_{0}\left(\beta |\kappa_{1},\ldots ,\kappa_{L}\right)=\sum_{l=1}^{L}\kappa_{l}𝕀(\beta ={\hat{\beta }}_{l}),$$

where $\kappa_{l}=p\left(\beta ={\hat{\beta }}_{l}\right)$. Further defining $\phi_{l}^{\left(i-1\right)}=p\left(\beta ={\hat{\beta }}_{l}|x_{-i},z_{-i}\right)$ and $\pi_{ikl}=p\left(z_{i}=k|\beta ={\hat{\beta }}_{l},z_{-i}\right)$, the *β* parameter may be marginalised in Equation (4) to obtain:

(7) $$p\left(z_{i}=k|x_{-i},x_{i},z_{-i}\right)=\frac{\sum_{l=1}^{L}\phi_{l}^{\left(i-1\right)}\pi_{ikl}L_{ik}\left(x_{i}\right)}{\sum_{l=1}^{L}\phi_{l}^{\left(i-1\right)}\sum_{k=1}^{K}\pi_{ikl}L_{ik}\left(x_{i}\right)},$$

where $\pi_{ikl}:=p\left(z_{i}=k|\beta ={\hat{\beta }}_{l},z_{-i}\right)$ is given by Equation (3); $\phi_{l}^{\left(0\right)}=\kappa_{l}$ and:

(8) $$\phi_{l}^{\left(i\right)}=p\left(\beta ={\hat{\beta }}_{l}|x_{-i},x_{i},z_{-i},z_{i}\right)=\frac{\phi_{l}^{\left(i-1\right)}\pi_{iz_{i}l}}{\sum_{s=1}^{L}\phi_{s}^{\left(i-1\right)}\pi_{iz_{i}s}}$$

may be calculated sequentially for *i* = 1, …, *n*. The SUGS algorithm for allocating observations to clusters when *β* is unknown is then as presented in Algorithm 1.

**Algorithm 1: The SUGS algorithm, when the DP precision parameter *β* is allowed to be unknown.**

  **Input**: Data $X={\left\{x_{i}\right\}}_{i=1}^{n}$, Prior *P*_0_(*θ*),Hyperparameters ${\left\{\kappa_{l}\right\}}_{l=1}^{L}$

  **Output**: Cluster allocations $Z={\left\{z_{i}\right\}}_{i=1}^{n}$

**1** Initialise *z*_1_ = 1, *K* = 2, and ${\left\{\phi_{l}^{\left(0\right)}=\kappa_{l}\right\}}_{l=1}^{L}$

**2** Evaluate ***p*(*θ*_1_|*z*_1_, *x*_1_)** ∝ *p*_0_(*θ*_1_)***f***(***x***_1_|*θ*_1_);

**3** Calculate ${\left\{\phi_{l}^{\left(1\right)}\right\}}_{l=1}^{L}$ according to Eq. (8);

**4 for** *i* = 2 to ***N* do**

**5     for** *k*= 1 *to K* **do**

**6**         Calculate *L_ik_* according to Eq. (5);

**7**         Evaluate *p*(*z_i_* = *k*|*x*_1_, …, *x_i_*, *z*_1_, …, *z*_*i*−1_) according to Eq. (7);

**8**     **end**

**9**     Set *z_i_* =arg max_*k*=1, …_, *K*(*p*(*z_i_* = *k*|*x*_1_, …, *x_i_*, *z*_1_, …, *z*_*i*−1_));

**10**     Set *K* = max{*z*_1_, …, *z_i_*} + 1;

**11**     for *l* = 1 *to L* **do**

**12**         Calculate $\phi_{l}^{\left(i\right)}$, according to Eq. (8);

**13**     **end**

**14**     Evaluate *p*(*θ_zi_*|*x*_1_, …, *x_i_*,*Z_1_*, …, *x_i_*,*z*_1_, …, *z*_*i*_) ∝ *p*_0_(*θ_z_i__*) ∏_*j*:*z_j_*=*z_i_*,1≤*j*≤*i*_ *f*(*x_j_*|*θ_z_i__*)

**15 end**

#### 2.2.2 Formulation of Bayesian model selection problem

A notable limitation of the (deterministic) SUGS algorithm as presented so far is that the clustering structure obtained is dependent upon the initial ordering of the observations. To remove this dependence, Wang and Dunson (2011) consider multiple permutations of this ordering, and pose SUGS as a Bayesian model selection (BMS) problem. More concretely, the algorithm is repeated for many random orderings of the data and a final partition of the observations is then chosen by optimising an appropriate objective function for BMS, such as the marginal likelihood (ML):

(9) $$L(X|Z)=\prod_{k=1}^{K}\underset{\theta_{k}}{\int }\left[\prod_{i:z_{i}=k}f\left(x_{i}|\theta_{k}\right)\right]p_{0}\left(\theta_{k}\right)d\theta_{k}.\;$$

In practice, Wang and Dunson (2011) advocate optimising the *pseudo-marginal* likelihood (PML), since they found that the marginal likelihood to often produce many small clusters. The PML is given by:

(10) $$\begin{array}{ll} PML_{z}(X) & =\prod_{i=1}^{N}p\left(x_{i}|X_{n\setminus -i},z_{n\setminus -i}\right)\\ =\underset{\theta }{\prod_{i=1}^{N}\int p\left(x_{i}|\theta \right)p\left(\theta |X_{n\setminus -i},z_{n\setminus -i}\right)d\theta }\\ \begin{array}{l} =\prod_{i=1}^{N}\sum_{k=1}^{K}P\left(z_{i}=k|X_{n\setminus -i},z_{n\setminus -i}\right)\underset{\theta_{k}}{\int }f\left(x_{i}|\theta_{k}\right)p\left(\theta_{k}|X_{n\setminus -i},z_{n\setminus -i}\right)d\theta_{k^{\prime }} \end{array} \end{array}$$

where, defining *X* = {*x*_1_, …,*x_n_*} and *Z* = {*z*_1_, …,*z_n_*}, we have *X*_*n*\−*i*_ = *X*\{*x_i_* is the set of all observations except the *ith*, and similarly *z*_*n*\−*i*_ = *Z*\{*z_i_*}. In addition, Wang and Dunson (2011) remark that that *p*(*x_i_*|*X, Z*) can be used to approximate *p*(*x_i_*|*X*_*n*\−*i*_, *z_*n*\−i_*) to speed up computations and that this approximation is accurate for large sample sizes.

#### 2.3 SUGS for variable selection

Irrelevant variables in high-dimensions can present a considerable challenge for clustering models and algorithms, because the number of variables with no clustering structure can overwhelm those where a clustering structure exists (Witten & Tibshirani, 2010). There have been many approaches to model-based clustering and variable selection (e.g. Raftery & Dean, 2006; Maugis, Celeux & Martin-Magniette, 2009), and we direct readers to Fop and Murphy (2018) for a recent review. However, many of these scale poorly with increasing dataset dimension, and/or require the number of clusters to be determined as a separate analysis step. To address these challenges, here we extend the SUGS algorithm to simultaneously perform clustering and variable selection, and refer to the resulting procedure as *SUGSVarSel*.

Since we are in the high-dimensional setting, we assume for simplicity that variables are independent given the cluster allocations (which, in the Gaussian case, is equivalent to assuming a diagonal structure for the covariance matrix). Let *x_i,d_* be the *dth* element of the *ith* observation vector, with *d* = 1, …, *D*, and *D* the number of variables. Introducing indicator variables *γ_d_*, which is 1 if the *dth* variable is relevant for the clustering structure and 0 if not, we follow a common approach from the literature (Law, Figueiredo & Jain, 2004; Tadesse, Sha & Vannucci, 2005; Kim, Tadesse & Vannucci, 2006) and assume that the cluster conditional likelihood can be factorised as follows:

(11) $$f\left(x_{i}|\theta ,\theta_{0},z_{i}=k\right)=\prod_{d=1}^{D}f{\left(x_{i,d}|\theta_{k,d}\right)}^{𝕀\left(\gamma_{d}=1\right)}f{\left(x_{i,d}|\theta_{0,d}\right)}^{𝕀\left(\gamma_{d}=0\right)},$$

where *θ*_0_ are “global” (i.e. not cluster-specific) parameters. In other words, the variables for which *γ_d_* = 1 are modelled by a mixture distribution with cluster-specific parameters *θ_k,d_*, while the variables for which *γ_d_* = 0 are modelled by a single component with (global, not cluster-specific) parameters *θ*_0,*d*_. Having introduced the *D* indicator variables *γ_d_*, we now extend the SUGS algorithm in order to estimate them.

#### 2.3.1 The SUGSVarSel algorithm

Given a realisation of the indicator variables, **Γ** = {*γ*_1_, …, *γ_D_*}, we may plug the cluster conditional likelihood given in Equation (11) into Equation (5) and proceed as before in order to identify a clustering, *Z*.

Conversely, suppose we have a realisation, *Z*, of the set of component allocation variables, but that the indicator variables Γ are unknown. In this case, the posterior probabilities associated with the variable indicators are given by:

(12) $$P\left(\gamma_{d}=1|X,Z\right)=\frac{p_{0}\left(\gamma_{d}=1\right)}{B}\prod_{k\in Z}\underset{\theta_{k,d}}{\int }\left(\prod_{i:z_{i}=k}f\left(x_{i,d}|\theta_{k,d}\right)\right)p_{0}\left(\theta_{k,d}\right)d\theta_{k,d}$$

(13) $$P\left(\gamma_{d}=0|X,Z\right)=\frac{p_{0}\left(\gamma_{d}=0\right)}{B}\underset{\theta_{0,d}}{\int }\left(\prod_{i:z_{i}=k}f\left(X_{d}|\theta_{0,d}\right)\right)p_{0}\left(\theta_{0,d}\right)d\theta_{0,d,}$$

where *p*_0_(*γ_d_* = *q*) indicates the prior probability that *γ_d_* = *q*, and *B* is a normalising constant that ensures that *P*(*γ_d_* = 0|*X*,*Z*) and *p*(*γ_d_* = 1|*X*,*Z*)sum to 1. Thus, given a realisation, *Z*, of the set of component allocation variables, a greedy approach to finding *γ_d_* is to set *γ_d_* = arg max_*q*∈{0,1}_ *P*(*γ_d_* = *q*|*X, Z*).

Given an initial realisation of the indicator variables, Γ= Γ^(0)^, the above suggests an iterative strategy in which at each iteration we use the SUGS algorithm to find a partition *Z*^(t)^ given Γ^(*t*−1)^, and then greedily update the indicator variables according to Equations (12) and (13) above in order to obtain Γ^(*t*)^ given *Z*^(t)^. This algorithm, which we refer to as SUGSVarSel, is presented in Algorithm 2.

**Algorithm 2: The SugsVarSel algorithm**

**Input**: Data $X={\left\{x_{i}\right\}}_{i=1}^{n}$, Priors *P*_0_(*θ*) and *P*_0_(*y*),

  Hyperparameters ${\left\{\kappa_{l}\right\}}_{l=1}^{L}$ Initial Indicator Switches Γ^(0)^

  Maximum Iterations *T*.

**Output**: Cluster allocation $Z={\left\{z_{i}\right\}}_{i=1}^{n}$ Variable switches $\Gamma ={\left\{y_{d}\right\}}_{d=1}^{D}$

**1** Initialise *z*_1_ = 1, *K* = 2, and ${\left\{\phi_{l}^{\left(0\right)}=\kappa_{l}\right\}}_{l=1}^{L}$;

**2** Evaluate *p*(*θ*_1_|*z*_1_, *x*_1_) ∝ *P*_0_(*θ*_1_)*f*(*x*_1_|*θ*_1_);

**3** Calculate ${\left\{\phi_{l}^{\left(1\right)}\right\}}_{l=1}^{L}$, according to Eq. (8);

**4 while** *t* ≤ *T* **do**

**5     for** *i* = 2 to *N* **do**

**6         for** *k* = 1 *to* *K* **do**

**7**            Calculate *L_ik_* given Γ^(*t*−1)^ according to Eqs. (5) and (11);

**8**            Evaluate *p*(*z_i_* = *k*|*x*_1_, …, *x_i_*, *Z*_1_, …, *z*_*i*−1_) according to Eq. (7);

**9         end**

**10**         Set *Z_i_* = arg max_*k*=1,…,*k*_(*p*(*z_i_* = *k*|*x*_1_, …, *x_i_*, *Z*_1_, …,*z*_*i*−1_));

**11**         Set *K* = max{*Z*_1_, …,*z*_*i*_} + 1;

**12         for** *l* = 1 *to L* **do**

**13**            Calculate $\phi_{l}^{\left(i\right)}$ according to Eq. (8);

**14         end**

**15**         Evaluate, using the cluster conditional likelihood in Eq. (11), *p*(*θ_z_i__*|*x_i_*,*z*_1_, …*z_i_*) ∝ *p*_0_(*θ_z_i__*) ∏_*j*:*z_j_*=*z_i_*,1≤*j*≤*i*_ *f*(*x_j_*|*θ_z_i__*);

**16     end**

**17     for** *d* = 1 *to* *D* **do**

**18**         Calculate ***p*(*y_d_* = *r*|*X*,*Z***), according to Eqs. (12) and (13);

**19**         Set *y_d_* = arg max_*r*∈{0,1}_(***p*(*y_d_* = *r*|*X*, *Z***));

**20     end**

**21** *t* ← *t* + 1

**22 end**

#### 2.3.2 Initialisation strategies for SUGSVarSel

Like the SUGS algorithm, the output of SUGSVarSel depends upon the initial ordering of the observations. It moreover depends upon the initialisation of the variable selection switches, **Γ^(0)^**. To address this latter issue, we propose a random sub-sampling initialisation strategy. This is as follows: first randomly select *p*_1_ variables (with 1 < *p*_1_ ≤ *D*) and apply SUGSVarSel on this new dataset $\tilde{X}$ of size *n*×*p*_1_ with a small number of random orderings of the observations (we find 10 works in practice). The initial indicator for the variables of $\tilde{X}$, which we write as ${\tilde{\Gamma }}^{\left(0\right)}$, are set as all-on (*γ_d_* = 1 for these *p*_1_ variables). ${\tilde{\Gamma }}^{\left(0\right)}$ is held the same for each of the random orderings. For each of the random orderings, this approach outputs $\tilde{Z}$ for all observations but $\tilde{\Gamma }$ for only a subset of size *p*_1_ of the variables. To obtain **Γ** for all *D* variables, we use the cluster allocations $\tilde{Z}$ and the full data *X* to compute probabilities for the remaining variables using 12 and 13. We then greedily assign the indicator variables. A single best model generated by these random orderings is selected using the ML. This procedure returns a Γ_1_ ∈ {0, 1}^*D*^; that is, variable selection switches with some variables switched on and other variables switched off. We repeat this process for a total of *M* random sub-samples of the variables to produce a set of clusterings *Z*_1_, …, *Z_M_* and a set of variables Γ_1_,… Γ_*M*_. These variable sets are then used as initial inputs Γ^(0)^ = Γ_i_ for *i* = 1, …, *M* for the SUGSVarSel algorithm (which is now run using all variables *p* = *D*) with *Q* new random orderings (again we find 10 is sufficient in practice). This SUGSVarSel with sub-sampling initialisation strategy returns *Q* models for each random sub-sample of the variables. Thus, we have *QM* models from which to choose. For each model obtained in this way, we calculate the marginal likelihood (see Section 2.2.2). We can then perform BMS to obtain a single “best” model, or we can use Bayesian model averaging (BMA; see next section).

### 2.4 Bayesian model-averaged co-clustering matrices

#### 2.4.1 Bayesian model averaging

The output of our algorithm is a set of clusterings, associated variables and a marginal likelihood. One can select a single *“*best*”* model amongst these possible clustering, however we can also average over these models to capture the model uncertainty. The idea is called Bayesian model averaging (BMA) and we apply the method to clustering and variable selection (Madigan & Raftery, 1994; Hoeting et al., 1999; Russell, Murphy & Raftery, 2015).

For each model we form a co-clustering matrix *S. S* is defined in the following way:

(14) $$S_{ij}=\{\begin{array}{ll} 0, & \text{if }z_{i}\neq z_{j}\\ 1, & \text{if }z_{i}=z_{j}. \end{array}$$

That is the *ijth* entry of *S* is 1 if observation *x_i_* and *x_j_* are in the same cluster and 0 otherwise. We note that the *S* is invariant to relabelling and the number of clusters. Now, suppose we have *M* models ℳ_1_, …, ℳ_*M*_, letting *X* be our observations and *θ_m_* be the parameters associated with model ℳ*_m_*. The posterior probability for ℳ*_m_* is given by

(15) $$p\left(M_{m}|X\right)=\frac{p\left(X|M_{m}\right)p_{0}(M)}{\sum_{l=1}^{M}p\left(X|M_{l}\right)p_{0}\left(M_{l}\right)},$$

where

(16) $$P\left(X|M_{m}\right)=\int P\left(X|\theta_{m},M_{m}\right)P\left(\theta_{m}|M_{m}\right)d\theta_{m}.$$

The marginal likelihood (16) is the key quantity for model comparison and can be interpreted as the weight given to each proposed model. Further note the two sources of averaging: the averaging over the parameters in the ML and the averaging over the models in equation (15). We suppose that *a priori* all models are equally likely, choosing the prior on each model to be *p*_0_(*ℳ_m_*) = 1/*M*. One computational challenge that (15) gives us is computing the summation, since it can involve evaluating possibly thousands of models. To overcome this, one can discount models that are poor at describing our observations comparatively to our best model. More precisely, let us form Occam’s window (Hoeting et al., 1999):

(17) $$W=\left\{M_{k}:\frac{\underset{l}{\max}\left(p\left(M_{l}|X\right)\right)}{p\left(M_{k}|D\right)}\leq K\right\},$$

where *K* is a tuning parameter. Occam’s window is the set of all possible models within a reasonable Bayes factor from the best model under consideration. The summation in (15) is then replaced with a summation over the set *𝒲*.

#### 2.4.2 Averaging the co-clustering matrices

We can form the Bayesian model-averaged co-clustering matrix (BMAC) by taking the set of co-clustering matrices *S_𝒲_* and averaging, weighting by their ML:

(18) $$S_{BMAC}=\frac{p\left(X|M_{m}\right)S_{m}}{\sum_{l\in W}p\left(X|M_{l}\right)}.$$

The BMA of the variable set can be found in the same way by averaging over the weighted variable sets for each model:

(19) $$F_{BMA}=\frac{p\left(X|M_{m}\right)F_{m}}{\sum_{l\in W}p\left(X|M_{l}\right)},$$

where we denote by *ℱ_m_* the variable set associated with model ℳ*_m_*.

## 3 Comparisons with the state-of-the-art

We compare sugsVarSel to a number of alternative algorithms, and demonstrate the performance of our method in two situations. The first is the *p* > *n* paradigm, where the number of variables exceeds the number of observations. The second situation considers *n* > *p* for *n* = 1000, while simultaneously considering different proportions of variables being relevant. In both cases, we consider a variety of scenarios, for which different proportions of the variables are relevant.

### 3.1 Alternative methods for clustering and variable selection

We compare our method relative to the current state-of-the-art, including methods that do and do not peform variable selection. These include: mclust, a finite mixture model based clustering method (Fraley & Raftery, 2002; Fraley et al., 2012; Scrucca et al., 2016); DP-means, a non-parametric interpretation of K-means (Kulis & Jordan, 2012); clustvarsel, a finite mixture model method with variable selection (Raftery & Dean, 2006; Maugis, Celeux & Martin-Magniette, 2009; Scrucca & Raftery, 2014); the original sequential updating and greedy search algorithm (Wang & Dunson, 2011) as implemented in our sugsvarsel R package; and VarSelLCM, a model-based clustering and variable selection approach using the integrated complete-data likelihood (Marbac & Sedki, 2017).

### 3.2 High-dimensional example

In the first example, we simulate a mixture of 3 Gaussians with mixture proportions 0.5, 0.3, 0.2 centred at (0,0,..,0), (2,2, …,2), (−2,−2,…,−2) respectively, each with variance-covariance matrix equal to the identity. The irrelevant variables are simulated from a standard Gaussian. First, we simulate 100 observations from this model with 200 variables and explore varying the number of relevant variables.

When running SUGS and SUGSVarSel we use the same prior specification for both methods and 30 random orderings of the data. Throughout this article, we always perform 2 iterations of variable selection in the SUGSVarSel algorithm. To initialise variable selection in SUGSVarSel, we subsample 10% of the variables 20 times to produce an initial variable selection set. For SUGS we choose the partition with maximal PML (as advised in the original SUGS paper by Wang and Dunson 2011), while for SUGSVarSel we select the result with maximal ML. Prior choices for SUGS and SUGSVarSel can be found in the Supplementary Material. For mclust and clustvarsel, we find the appropriate number of clusters using a sequential search up to a maximum of 9 possible clusters. We then use then Bayesian Information Criterion (BIC) to select an appropriate model (Schwarz, 1978). For DP-means we repeat the algorithm over a range of penalty parameters *λ* ={0.01, 0.1,1,10, 100, 200, 400, 600, 800, 1000} and select the partition which minimises the DP-means objective function. For VarSelLCM we run the algorithm up to a maximum of 9 possible clusters and select an appropriate model using the Maximum Integrated Complete-data Likelihood (MICL) (Marbac & Sedki, 2017, 2018). All methods are run in serial for fair comparison.

Results are presented in Table 1–Table 4. In all tables, we provide runtimes for each of the methods, indicate the proportion of relevant and irrelevant variables that each method correctly identified (for methods without variable selection this is reported as 1 for relevant and 0 for irrelevant variables), and report the adjusted Rand index (Rand, 1971; Hubert & Arabie, 1985) between the clustering produced and the truth. We repeat all methods for 10 different random realisation of the datasets to produce a distribution of scores. We report the median scores, along with the upper and lower quartiles.

It is evident that methods that do not perform variable selection such as mclust and SUGS perform poorly when there are many irrelevant variables. The performance of clustvarsel here seems volatile and performs poorly at correctly selecting relevant features. VarSelLCM and SUGSVarSel are competitive in terms variable selection and clustering. However, VarSelLCM requires an exhaustive search over the number of clusters, which makes this method computationally costly to apply when the number of clusters is not known. SUGSVarSel outperforms all variable selection and clustering methods in terms of speed, while also automatically inferring the number of clusters in the data. We proceed to evaluate the performance of SUGSVarSel on large simulated datasets.

#### 3.2.1 Increasing the number of observations

We simulate the same distribution as before, but instead sample 1000 observations and only 100 variables and the irrelevant variable are simulated from a standard Gaussian distribution. All priors are the same as in the previous analysis and we sub-sample 10% of the variables 10 times to produce an initial variable selection set. We repeat SUGS and SUGSVarSel for 10 random orderings of the data. We compare the scalable methods mclust, DP-means, SUGS, SUGSVarSel and VarSelLCM, where 25%, 10%, 5% of the variable are relevant. For SUGS we choose the partition with maximal PML, while for SUGSVarSel we select the result with maximal ML. For VarSelLCM we run the algorithm for possible number of clusters 1 through 4 and select an appropriate model using the MICL, as previously. Results are presented in Table 5–Table 7.

Mclust, SUGS and DP-means produce poor quality clusterings, because irrelevant variables present in the data render finding the true underlying clustering structure challenging. SUGSVarSel and VarSelLCM produce high quality answers in all situations but SUGSVarSel is 2 orders of magnitude faster. However, to alleviate the computational burden we searched up to a maximum of 4 clusters in VarSelLCM, providing it with an easier opportunity to produce high quality clusterings. In applications to real data this would have to be much larger, adding considerably to computational time, whereas the inference of the number of clusters is automatic in SUGSVarSel.

### 3.3 Advantages of Bayesian model averaging

**Figure 1:**
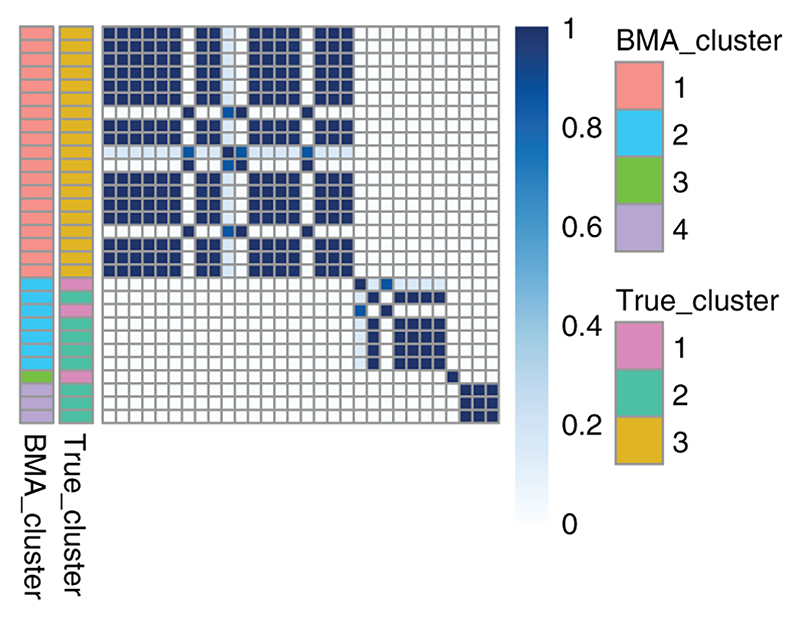
A heatmap of the BMA co-clustering matrix, where dark blue indicates the probability of being in the same cluster is 1 and white indicates a probability of 0 of belonging to the same cluster. The component annotation bar indicates the true component labels and the cluster annotation bar indicates the clustering obtained from summarising the BMA co-clustering matrix.

As an example, we simulate a dataset with 30 observations from a mixture of 3 Gaussians, where two of the Gaussians are isotropic and centred (2, 2) and (−3, −3), respectively, each with mixing weights 0.4. The third component has mixture weight 0.2 and is centered at (−3, 4) but the covariance matrix is 2 on the diagonals and 1 on the off diagonals, violating our independence assumption. We additionally include 2 components of irrelevant variables generated from standard Gaussians. Our prior specifications are set as in the previous section. Simply using the ML to pick a partition results in an ARI of 0.635 between the clustering produced and the truth. However, we can also perform BMA and then summarise our co-clustering. We applied hierarchical clustering with average linkage to compute a clustering, which has previously be applied to posterior similarity matrices (Medvedovic, Yeung & Bumgarner, 2004; Fritsch & Ickstadt, 2009; Liverani et al., 2015) (see Supplementary Material for complete details). This clustering then produces an ARI of 0.875. The heatmap of the co-clustering matrix is plotted in Figure 1, allowing us to visualise the uncertainty in the clustering.

## 4 Applications to cancer subtyping

### 4.1 Application to leukaemia dataset

In this section, we apply SUGSVarSel to real biological datasets. The first is a well-studied genomic clustering problem: the separation of acute myeloid leukaemia (AML) and the B/T-cell subtypes of acute lymphoblastic leukemia (ALL) samples on the basis of microarray transcriptomic data. We use the dataset described by Golub et al. (1999), which comprises 38 samples, 27 of which are ALL (8 T-cell and 19 B-cell related), and 11 of which are AML cases. Initial preprocessing is performed as in Dudoit, Fridlyand, and Speed (2002), which reduces the dimension of the dataset from 6817 to 3051 genes. In Dudoit, Fridlyand, and Speed (2002), a further dimension reduction step is performed that makes use of the AML and ALL class labels, so that only those genes that have a high ratio of their between-class to within-class sums of squares are retained. Here we instead wish to adopt a completely unsupervised approach, so that we may use the known ALL-AML class label in order to validate our results.

We select the 200 most variable genes and then normalise, so the expression values for each gene are mean-centred at 0 with variance 1. 200 genes were chosen because this led to good predictive performance in previous analysis of these data (Golub et al., 1999; Dudoit, Fridlyand & Speed, 2002). We then apply SUGSVarSel to the resultant dataset. We sub-sample 10% of the variables 20 times to produce an initial variable selection set, and run the algorithm for 100 random orderings. We adopt our default priors and summarise the output using BMA. A final summary clustering is obtained by performing hierarchical clustering with average linkage (Fritsch & Ickstadt, 2009). We use the ARI to compare our results to the truth (of 3 classes) and repeat the process 10 times and report the average results.

**Figure 2:**
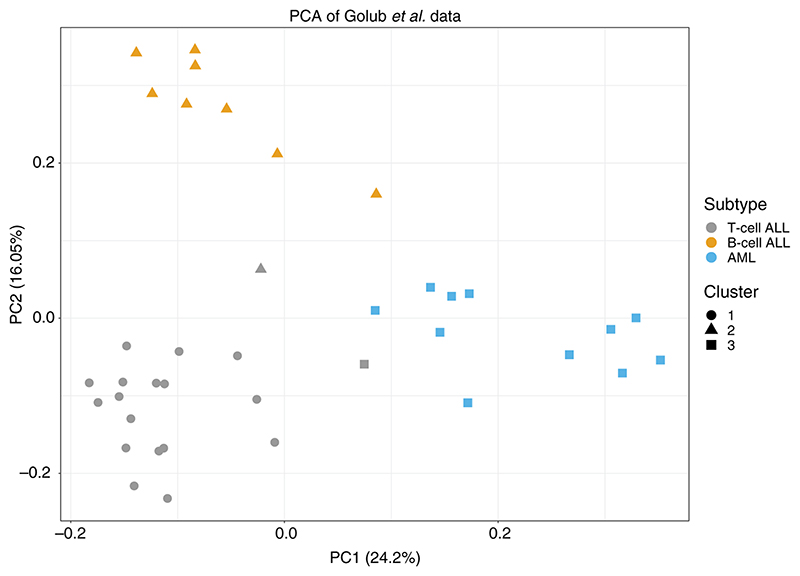
A PCA plot of the microarray expression data of 38 patients from the Golub et al. (1999) dataset, using the 200 most variable genes. The different symbols indicate the clustering produced by the SUGSVarSel algorithm after summarising the BMA co-clustering matrix using hierarchical clustering with average linkage. The colours indicate the annotated sub-types.

Results are illustrated in Figure 2. The final clustering result provides an ARI of 0.831, which is in line with previous analyses preformed on this dataset (Golub et al., 1999; Dudoit, Fridlyand & Speed, 2002). The algorithm selects a total of 92 genes, including TCL1, TCRB, IL8, EPB72, IL7R, TCRG, NFIL6, which are all known to be associated with leukaemia (Natsuka et al., 1992; Pekarsky, Hallas & Croce, 2001; Van der Velden et al., 2004; Kuett et al., 2015; Chen, Tsau & Lin, 2010; Shochat et al., 2011). A full list of the selected genes (including their descriptions) can be found in the Supplementary Material. The advantage of our analysis over other methods is that we did not need to specify the number clusters – the algorithm automatically inferred 3 clusters in the data, which have excellent correspondence to the known classes of AML and ALL, as well as the 2 ALL subgroups.

**Figure 3:**
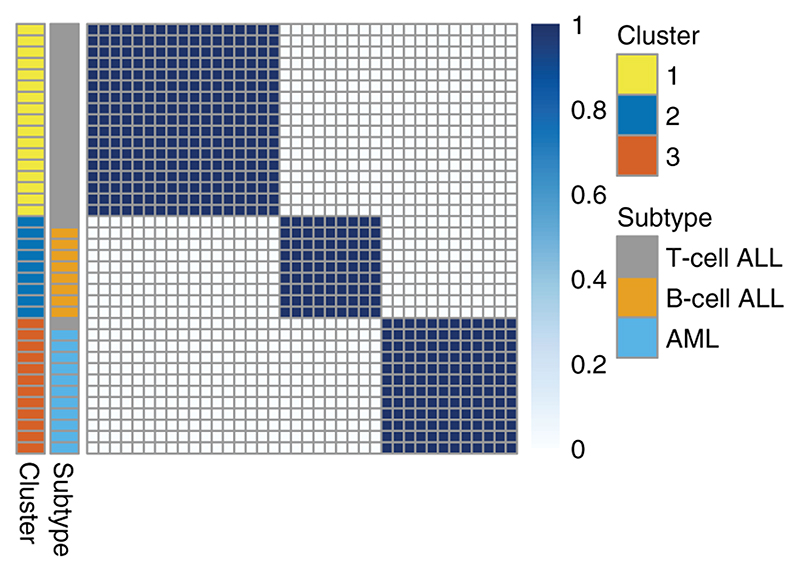
A heatmap of the BMA co-clustering matrix for the 38 patients, when applying SUGSVarSel, demonstrating the added benefit of visualising uncertainty. The annotation bars of the left indicate the correspondence between the clusters and the subtypes.

To assess the importance of variable selection, we also apply mclust and the original SUGS algorithm to the data. We run the mclust algorithm performing a systematic search to select the number of clusters, up to a maximum of 9, and select the number of cluster which maximises the BIC. This criterion selects 3 clusters and clustering produced gives an adjusted Rand index of 0.627 – the inclusion of irrelevant variables has led to reduced cluster quality. We run SUGS using our default prior choices and using the PML criterion to select a clustering. The algorithm was run for 100 random ordering and we repeated the process 10 times, reporting an average ARI of 0. The lack of variable selection renders SUGS unable to produce a meaningful clustering. In Figure 3, we visualise the BMA co-clustering matrix for these data when applying the SUGSVarSel algorithm.

### 4.2 Application to TCGA breast cancer dataset

We demonstrate SUGSVarSel on a further genomics dataset. We analyse an expression dataset for breast cancer tumour data from The Cancer Genome Atlas (TCGA) (Network, 2012), which we pre-process in the same way as in Lock and Dunson (2013). The processed expression dataset comprises 348 tumours with 645 genes, of which 14 belong to the PAM50 (Prediction Analysis of Microarray) group of genes (Parker et al., 2009).

Analysis was performed in the following way. We first standardise our data so that each column is mean-centred with variance 1. We then subsample 10% of the variables 64 times to produce an initial variable set. We then apply the SUGSVarSel algorithm with default settings. We summarise our output by performing BMA and then hierarchical clustering with average linkage.

SUGSVarSel reveals two clusters in the dataset, the second of which is significantly associated with Basal-like tumours (Fisher test, *p* < 0.0001). The algorithm selects 245 variables to discriminate between the groups. We perform PCA before and after variable selection to demonstrate that the reduced variable set produces more separable and therefore more interpretable clusters. Furthermore, the algorithm selected 13 out of a total of 14 of the PAM50 genes, which is significantly better than random (Fisher Test, *p* < 0.0001).

There is perhaps concern that variable selection could remove relevant genes for clustering, in the situation where we have a highly informative set of variables. We consider the following task to cluster the breast cancer genes using the PAM50 genes from the total unprocessed dataset (that is without the filtering of Lock and Dunson (2013)), of which there are 48. We apply the SUGSVarSel in identical fashion to before, sub-sampling 10% of the variables 4 time to produce an initial variable set. We obtain 5 clusters which correspond well to the different breast cancer subgroups.

Cluster 1 is associated with Luminal A cancers, cluster B is associated with Luminal cancers, cluster 3 with basal-like tumours, cluster 4 contains mostly HER2 type breast cancers (chi-squared *p* < 0.0001). Thus, hardly surprisingly, the cluster produce on the PAM50 data coincide well with the PAM50 subgroups. Furthermore, 87.5% of the genes were selected which is more than we expect given our prior, telling us this was a highly informative set of genes.

**Figure 4:**
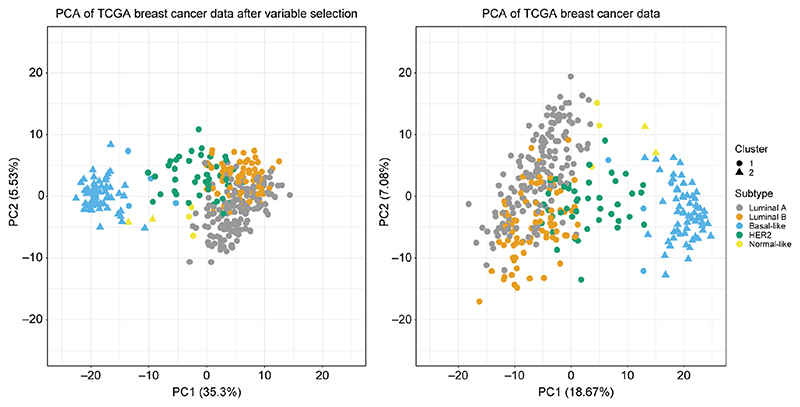
PCA plot on the TCGA breast cancer data, where clusters produced by SUGSVarSel are indicated by shape and subtypes by colour. The left PCA plot demonstrates smaller and tighter clusters using only the variables that remained after variable selection. In the right hand plot all variable were used to produce the plot.

**Figure 5:**
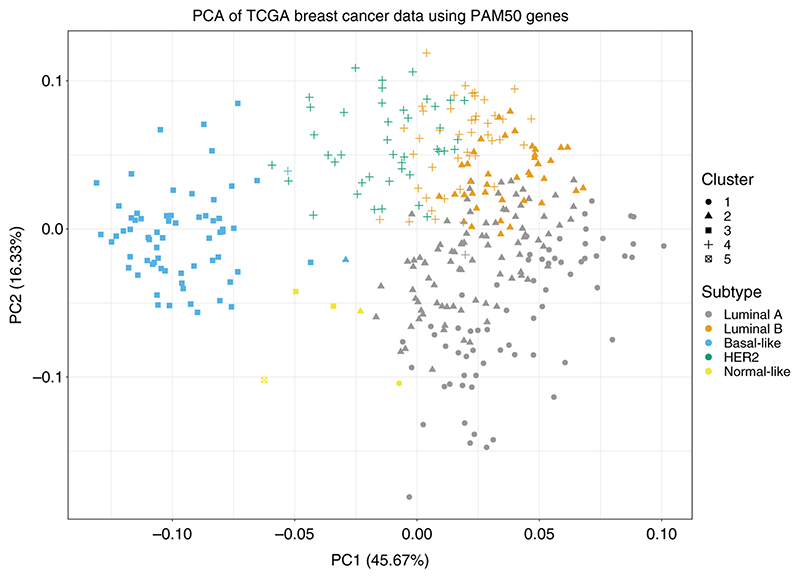
PCA plot on the TCGA breast cancer data using 48 of the PAM50 genes, where clusters produced by SUGSVarSel are indicated by shape and subtypes by colour.

The clusterings shown in Figure 4 and Figure 5 demonstrate that the variables we use for clustering are critically important. The two different pre-filtering choices led to results of varying quality and biological meaning. This is strong evidence in support of model-based variable selection rather than ad-hoc preprocessing.

## 5 Pan-cancer proteomic characterisation

In this section we apply our method to The Cancer Proteome Atlas (TCPA) datasets (Li et al., 2013; Akbani et al., 2014; Städler et al., 2017). The dataset contains a large number of tumours and cell line samples with protein expression levels generated using reverse-phase protein arrays (RPPAs). Our method allows us to perform a number of tasks on this data; in particular, for each cancer we can detect possible subgroups and the relevant proteins which discriminate these subgroups. We can also perform a pan-cancer analysis to explore the differences and similarities between cancers. Pan-cancer studies can unravel inter-cancer relationships which are important for developing new clinical targets (Weinstein et al., 2013; Uhlen et al., 2017; Berger et al., 2018; Hoadley et al., 2018). Recent pan-cancer analyses have suggested that cancers should be classified based on their molecular signatures rather than tissue of origin (Berger et al., 2018; Hoadley et al., 2018) and this motivates our analysis.

As is usual with this data there are irrelevant variables so methods that do not perform variable selection such as mclust and SUGS are ill-suited. Furthermore, there is little *a priori* knowledge about the number of clusters and so methods such as VarSelLCM and clustvarsel which require an exhaustive search of the number of clusters are inappropriate. To perform the analysis on all cancer sets would be prohibitively slow for the slowest of analysis methods.

The TCPA datasets contain data on 19 cancer types and the description of these cancers can be found in Supplementary Material. The total dataset consists of over 5000 tumour samples with only a few samples for some cancers and hundreds of samples for others and several hundreds of proteins. The merged PAN-Can 19 level 4 dataset is used in the following analysis, since it is appropriate for multiple disease analysis. More information about the data can be found here http://tcpaportal.org/tcpa/, where the data itself can also be downloaded. In addition, we standardise the expression levels for each protein so that they are zero-centred with unit variance.

The following table demonstrates the number of cases for each cancer type (Table 8).

**Figure 6:**
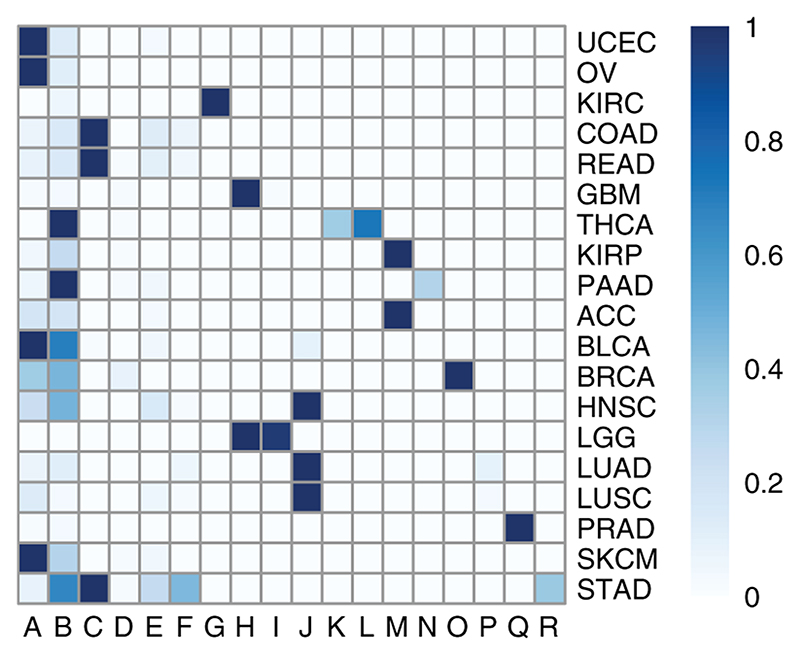
A heatmap indicating the correspondence between clusters produced by the SUGSVarSel algorithm and the different cancer types.

**Figure 7:**
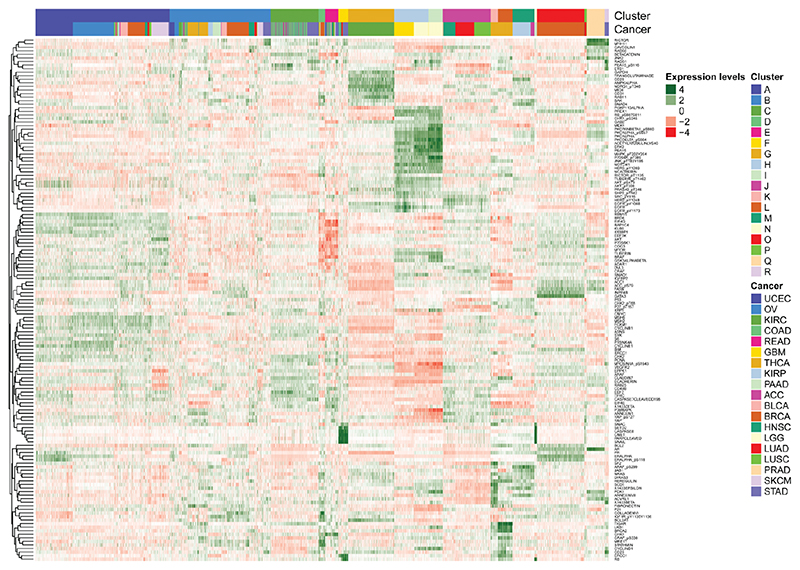
A heatmap of the expression data using the clustering produced by the SUGSVarSel algorithm applied to the pan-cancer TCPA dataset. The annotation bars on the top of plot indicate the different cancers and clusters.

We only keep proteins which have been measured on all cancers, which total 217 and so our dataset has a total of 5157 tumour samples with 217 variables. We apply SUGSVarSel to this data by first sub-sampling 10% of the variables 43 (a fifth of the total number of variables) times. Using the same priors as in previous analysis we analyses this data using the SUGSVarSel algorithm, running the algorithm for 50 random orderings, thus exploring a total of 2150 models. We summarise the BMA clustering using hierarchical clustering with average linkage. The summarised clustering contains 60 clusters, however many of these clusters contain only a few observations. Reassuringly there are 18 clusters with more than 20 observations and we focus on these for our analysis. A table summarising the clusters, along with results from hierarchical clustering, can be found in the Supplementary material, in Figure 6 is a heatmap of the clusterings:

In addition, we plot a heatmap of the data with the clustering produce by SUGSVarSel using only the proteins selected by the algorithm (Figure 7).

It is rare that a cancer associates with a single cluster, however there are evident relationships between cancers and clusters. Cluster A contains predominately womens’ cancers (OV, UCEC, BRCA), while cluster B contains a large spread of cancers. Clusters C, E and F contain the cancers of the digestive tract (STAD, COAD and READ). Cluster D contains a subgroups of breast cancers (BRCA), while cluster G contains solely kidney cancer (KIRC). Clusters H and I contain cancers of the brain (LGG, GBM). Cluster J and P contain aero-digestive cancers (HNSC, LUAD LUSC). Thyroid cancer (THCA) is spread across clusters K, L and B, whilst KIRP is predominately found in cluster M. Pancreatic cancer (PAAD) is split across clusters N and B. Cluster O contains the majority of breast cancer patients. Prostate cancer (PRAD) is dominantly found in Q, while R forms a small cluster of stomach cancers. This is in line with other analyses performed on these data (Akbani et al., 2014; Hoadley et al., 2014; Şenbabaoğlu et al., 2016). A total of 147 proteins were selected as relevant for clustering.

**Figure 8:**
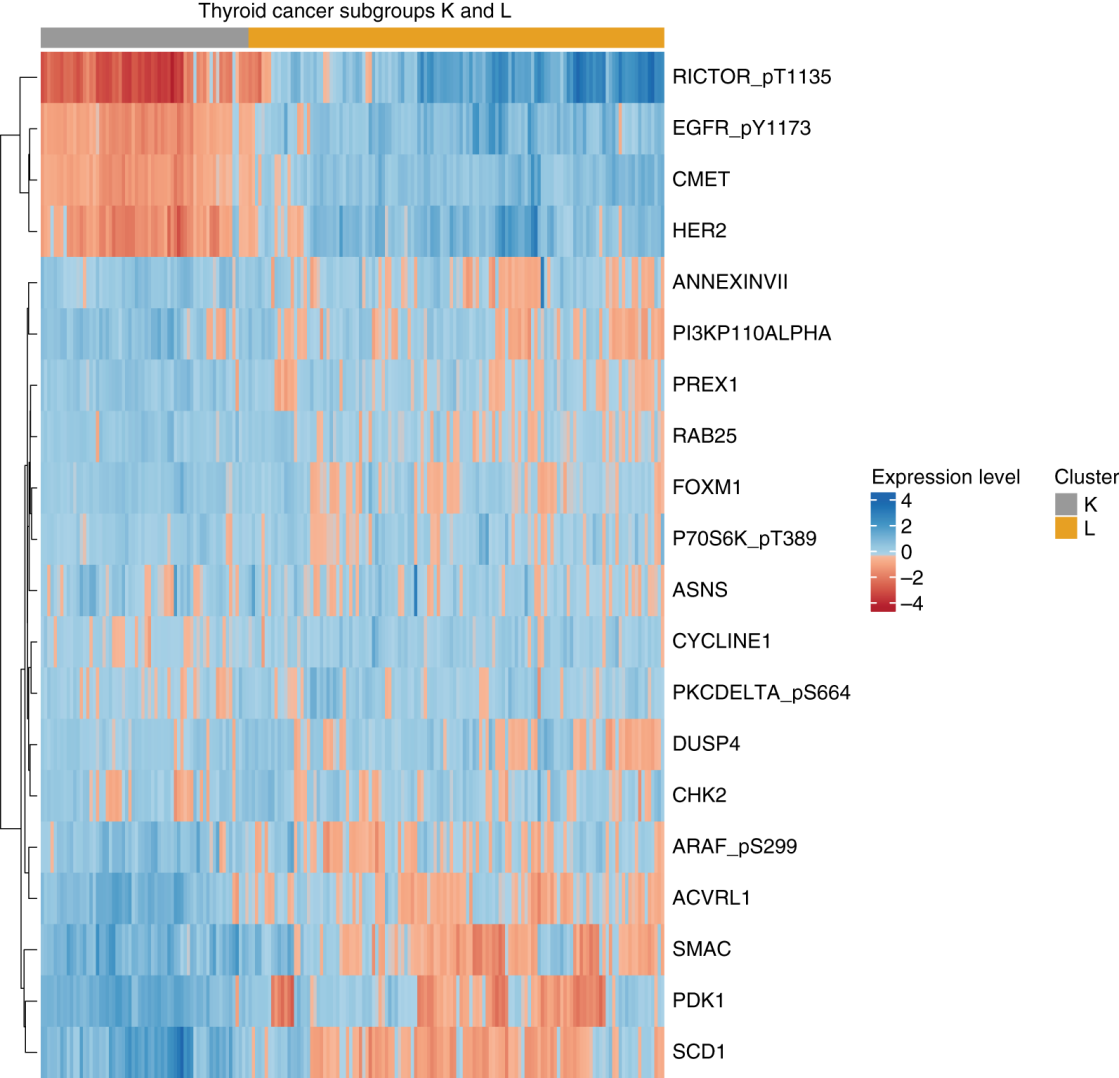
A heatmap of the expression TCPA data for the thyroid subgroups. We have plotted the expression for only the top 20 proteins which are significantly different between clusters K and L.

We now consider an illustrative example. Figure 6 shows us that clusters K and L contain only thyroid cancers. It is of biological interest to see what drives the differences between these clusters as they could define clinically relevant thyroid subgroups. Considering only the 147 selected proteins, we plot the expression profile for the 20 proteins (Figure 8), with smallest *p*-value, which are significantly different between clusters K and L (T-test (Welch, 1947), *p* < 0.00001, using Benjamini-Hochberg correction (Benjamini & Hochberg, 1995)).

We do not observe an over representation of any of the thyroid cancers subtypes within each of these clusters (see Table 9).

## 6 Conclusion

In this article we presented SUGSVarSel, an extension to the SUGS algorithm of Wang and Dunson (2011) to allow variable selection. We demonstrated that when irrelevant variables are present the quality of the clustering can be degraded and clusters become more challenging to interpret. SUGSVarSel allows the flexibility of a Bayesian nonparametric approach but inference is considerably faster than using MCMC. Indeed, the SUGSVarSel algorithm infers the number of clusters automatically and performs inference for the Dirichlet process hyperparameter. This is in contrast to most clustering with variable selection methods which require a systematic search over the number of clusters.

Whilst our method is approximate it performs competitively with other commonly used approaches. Furthermore, we take advantage of exploring many models by performing Bayesian model averaging, which is important for exploring uncertainty in our clustering. We remark that model uncertainty and the application of BMA is rarely explored in clustering tasks. We have provided an R package to facilitate dissemination of our method utilising C++ to accelerate intensive computations and parallel processing features to make further computational gains

Application to two cancer transcriptomic datasets show the clear benefit of simultaneously performing variable selection and clustering. We demonstrate that variable selection improves interpretation of these datasets, providing the genes that drive the clustering structure of the data, as well as identifying those that are irrelevant for clustering. We further applied our method to a pan-cancer proteomic dataset for which none of the current model-based clustering and variable selection methods are suitable. SUGSVarSel is able to provide a characterisation of 5157 tumour samples, demonstrating clustering relationships across cancer types based on their molecular signature rather the tissue of origin.

There are a number of ways in which our proposed method could be extended. Firstly, our assumption that variables are conditionally independent given the cluster allocations might be unrealistic for some datasets. In such cases, more elaborate variable selection methods might be desirable, although this is likely to come at increased computational cost. Furthermore, we have assumed conjugacy throughout, so that the marginal likelihood in Equation (5) may be evaluated analytically. As noted in the original SUGS paper of Wang and Dunson (2011), one possible way to extend to non-conjugate cases would be to approximate this marginal likelihood, e.g. using a Laplace approximation.

## Supplementary Material

Supplementary File

Supplementary File 1

## Figures and Tables

**Table 1:** High-dimensional simulation example where 100 observations are simulated from a Gaussian mixture distribution with 3 components and 200 variables, in which 50% of variables are relevant.

Method	Time, secs	Correct relevant variables	Correct irrelevant variables	ARI
mclust	<1	1	0	1[1, 1]
DP-means	<1	1	0	0.60 [0.37, 0.66]
clustvarsel	14280.8 [10431.6, 20310.4]	0.47 [0.45, 0.48]	1[1, 1]	1[1, 1]
SUGS	0.92 [0.90, 0.97]	1	0	0.955 [0.90, 0.97]
SUGSVarSel	24.6 [23.8, 24.9]	1[1, 1]	1[1, 1]	1[1, 1]
VarSelLCM	620.0 [574.9, 650.8]	1[1, 1]	1[1, 1]	1[1, 1]

**Table 2:** High-dimensional simulation example where 100 observations are simulated from a Gaussian mixture distribution with 3 components and 200 variables, in which 25% of variables are relevant.

Method	Time, secs	Correct relevant variables	Correct irrelevant variables	ARI
mclust	<1	1	0	1[1, 1]
DP-means	<1	1	0	0.74 [0.70, 0.79]
clustvarsel	1852.3 [1185.2, 5880.8]	0.02 [0.02, 0.02]	0.847 [0.812, 0.945]	0.01 [0.00, 0.04]
SUGS	2.07 [1.89, 2.16]	1	0	0.78 [0.72, 0.84]
SUGSVarSel	21.9 [21.9, 22.1]	1[1, 1]	1[1, 1]	1[1, 1]
VarSelLCM	487.7 [481.3, 494.1]	1[1, 1]	1[1, 1]	1[1, 1]

**Table 3:** High-dimensional simulation example where 100 observations are simulated from a Gaussian mixture distribution with 3 components and 200 variables, in which 10% of variables are relevant.

Method	Time, secs	Correct relevant variables	Correct irrelevant variables	ARI
mclust	<1	1	0	0 [0, 0]
DP-means	<1	1	0	0 [0, 0]
clustvarsel	3095.8 [2377.3, 3302.7]	0.05 [0.05, 0.10]	0.803 [0.778, 0.854]	0 [0, 0]
SUGS	5.02 [4.76, 5.23]	1	0	0.18 [0.13, 0.21]
SUGSVarSel	19.7 [19.5, 19.9]	1[1, 1]	1[1, 1]	1[1, 1]
VarSelLCM	523.5 [521.6, 532.0]	1[1, 1]	1[1, 1]	1[1, 1]

**Table 4:** High-dimensional simulation example where 100 observations are simulated from a Gaussian mixture distribution with 3 components and 200 variables, in which 5% of variables are relevant.

Method	Time, secs	Correct relevant variables	Correct irrelevant variables	ARI
mclust	<1	1	0	0 [0, 0]
DP-means	<1	1	0	0 [0, 0]
clustvarsel	2183.1 [802.5, 2992.4]	0.1 [0.1, 0.1]	0.879 [0.814, 0.959]	0 [0, 0]
SUGS	6.30 [6.07,10.11]	1	0	0.04 [0.02, 0.05]
SUGSVarSel	19.9 [19.7, 20.5]	1[1, 1]	1[1, 1]	1[1, 1]
VarSelLCM	583.5 [521.6, 532.0]	1[1, 1]	1[1, 1]	1[1, 1]

**Table 5:** Simulation example where 1000 observations are simulated from a Gaussian mixture distribution with 3 components and 100 variables, in which 25% of variables are relevant.

Method	Time, secs	Correct relevant variables	Correct irrelevant variables	ARI
mclust	11.2 [10.9, 11.6]	1	0	0 [0, 0]
DP-means	22.3 [21.7, 23.1]	1	0	0.30 [0.25, 0.36]
SUGS	3.4 [3.1, 3.6]	1	0	0.98 [0.97, 0.98]
SUGSVarSel	31.2 [30.7, 31.8]	1[1, 1]	1[1, 1]	1[1, 1]
VarSelLCM	3596.8 [2639.5, 7537.7]	1[1, 1]	1[1, 1]	1[1, 1]

**Table 6:** Simulation example where 1000 observations are simulated from a Gaussian mixture distribution with 3 components and 100 variables, in which 10% of variables are relevant.

Method	Time, secs	Correct relevant variables	Correct irrelevant variables	ARI
mclust	11.0 [10.7, 11.4]	1	0	0 [0, 0]
DP-means	21.4 [21.0, 21.8]	1	0	0.11 [0.02, 0.22]
SUGS	5.1 [4.9, 5.3]	1	0	0.01 [0.01, 0.04]
SUGSVarSel	33.3 [33.0, 33.8]	1[1, 1]	1[1, 1]	0.90 [0.80, 0.97]
VarSelLCM	1938.5 [1852.3, 1973.9]	1[1, 1]	1[1, 1]	0.997 [0.994, 0.997]

**Table 7:** Simulation example where 1000 observations are simulated from a Gaussian mixture distribution with 3 components and 100 variables, in which 5% of variables are relevant.

Method	Time, secs	Correct relevant variables	Correct irrelevant variables	ARI
mclust	11.4 [11.2,15.7]	1	0	0 [0, 0]
DP-means	22.0 [21.1, 22.7]	1	0	0 [0, 0]
SUGS	6.3 [5.6,11.1]	1	0	0 [0, 0]
SUGSVarSel	60.8 [59.8, 64.2]	1[1, 1]	1 [0.99, 1]	0.78 [0.54, 0.92]
VarSelLCM	2688.8 [2588.9, 2878.6]	1[1, 1]	1[1, 1]	0.943 [0.931, 0.945]

**Table 8:** A table indicating the different cancer types and the number of observations from each of those cancers.

ACC	BLCA	BRCA	COAD	GBM	HNSC	KIRC	KIRP	LGG	LUAD
46	127	820	327	205	203	445	208	257	234
**LUSC**	**OV**	**PAAD**	**PRAD**	**READ**	**SKCM**	**STAD**	**THCA**	**UCEC**	
192	411	105	164	129	207	299	374	404	

**Table 9:** A table showing the distribution of 3 different THCA subtypes across the clusters K and L produce from the SUGSVarSel algorithm.

	K	L
Thyroid papillary carcinoma – classical/usual	31	72
Thyroid papillary carcinoma – follicular (>= 99% follicular patterned)	17	25
Thyroid papillary carcinoma – tall cell (>= 50% tall cell features)	2	6

Note that this information was not available for all patients.
