# Supplementary material for: Fast approximate inference for variable selection in Dirichlet process mixtures, with an application to pan-cancer proteomics
Source: Stat Appl Genet Mol Biol. Author manuscript; Available in PMC 2023 Jan 3. (PMC7614016; doi:10.1515/sagmb-2018-0065)
Supplement: Supplementary File [file EMS158447-supplement-Supplementary_File.zip › j_sagmb-2018-0065_suppl/golubgene.pdf]

1 "TCL1 gene (T cell leukemia) extracted from H.sapiens mRNA for Tcell leukemia/lymphoma 1"
2 "TCRB T-cell receptor, beta cluster"
3 "INTERLEUKIN-8 PRECURSOR"
4 "TCRB T-cell receptor, beta cluster"
5 "GB DEF = MAL gene exon 4"
6 "Interleukin 8 (IL8) gene"
7 "GB DEF = (lambda) DNA for immunoglobin light chain"
8 "CST3 Cystatin C (amyloid angiopathy and cerebral hemorrhage)"
9 "CD24 signal transducer mRNA and 3' region"
10 "IGHM Immunoglobulin mu"
11 "MPO Myeloperoxidase"
12 "MHC class II HLA-DP light chain mRNA"
13 "GB DEF = Cystic fibrosis antigen mRNA"
14 "LTB Lymphotoxin-beta"
15 "Major Histocompatibility Complex, Class Ii Beta W52"
16 "CD9 CD9 antigen"
17 "MB-1 gene"
18 "DF D component of complement (adipsin)"
19 "PROBABLE PROTEIN DISULFIDE ISOMERASE ER-60 PRECURSOR"
20 "LGALS3 Lectin, galactoside-binding, soluble, 3 (galectin 3) (NOTE: redefinition of symbol)"
21 "LYZ Lysozyme"
22 "ANX1 Annexin I (lipocortin I)"
23 "IGB Immunoglobulin-associated beta (B29)"
24 "Azurocidin gene"
25 "Na,K-ATPase gamma subunit mRNA"
26 "CD1B CD1b antigen (thymocyte antigen)"
27 "TCF7 Transcription factor 7 (T-cell specific)"
28 "Mac25"
29 "PSAP Sulfated glycoprotein 1"
30 "Terminal transferase mRNA"
31 "CALGRANULIN A"
32 "CLASS II HISTOCOMPATIBILITY ANTIGEN, M ALPHA CHAIN PRECURSOR"
33 "SELL Leukocyte adhesion protein beta subunit"
34 "GB DEF = T-lymphocyte specific protein tyrosine kinase p56lck (lck) abberant mRNA"
35 "MEF2C MADS box transcription enhancer factor 2, polypeptide C (myocyte enhancer factor 2C)"
36 "PRG1 Proteoglycan 1, secretory granule"
37 "CD2 CD2 antigen (p50), sheep red blood cell receptor"
38 "CTGF Connective tissue growth factor"
39 "Lymphoid-restricted membrane protein (Jaw1) mRNA"
40 "GRO2 GRO2 oncogene"
41 "SEF2-1A protein (SEF2-1A) mRNA, 5' end"
42 "GB DEF = T-cell antigen receptor gene T3-delta"
43 "CYSTATIN A"
44 "NPY Neuropeptide Y"
45 "Amphiregulin (AR) gene"
46 "ELA2 Elastatse 2, neutrophil"

47 "SNRPN Small nuclear ribonucleoprotein polypeptide N"
48 "Adenosine triphosphatase, calcium"
49 "MHC cell surface glycoprotein (HLA-DQA) mRNA, 3'end"
50 "Zyxin"
51 "HU-K4 mRNA"
52 "GLUTATHIONE S-TRANSFERASE, MICROSOMAL"
53 "GB DEF = CD1 R2 gene for MHC-related antigen"
54 "GB DEF = Neutrophil elastase gene, exon 5"
55 "LYZ Lysozyme"
56 "Fc-epsilon-receptor gamma-chain mRNA"
57 "HLA-DRB1 Major histocompatibility complex, class II, DR beta 5"
58 "Lysozyme gene (EC 3.2.1.17)"
59 "LYZ Lysozyme"
60 "Pre-B cell enhancing factor (PBEF) mRNA"
61 "LPAP gene"
62 "CHIT1 Chitinase 1"
63 "TCRG T cell receptor gamma chain"
64 "MXS1 Membrane component, X chromosome, surface marker 1"
65 "FLN1 Filamin 1 (actin-binding protein-280)"
66 "BLK Protein-tyrosine kinase blk"
67 "DP2 (Humdp2) mRNA"
68 "HLA CLASS II HISTOCOMPATIBILITY ANTIGEN, DR ALPHA CHAIN PRECURSOR"
69 "Quiescin (Q6) mRNA, partial cds"
70 "GB DEF = Immunoglobulin mu, part of exon 8"
71 "ICAM3 Intercellular adhesion molecule 3"
72 "NF-IL6-beta protein mRNA"
73 "Nuclear Factor Nf-Il6"
74 "Protein-tyrosine-phosphatase (tissue type: foreskin)"
75 "DAGK1 Diacylglycerol kinase, alpha (80kD)"
76 "CD72 CD72 antigen"
77 "GB DEF = Selenoprotein W (selW) mRNA"
78 "PROBABLE G PROTEIN-COUPLED RECEPTOR LCR1 HOMOLOG"
79 "ARHG Ras homolog gene family, member G (rho G)"
80 "CA2 Carbonic anhydrase II"
81 "APLP2 Amyloid beta (A4) precursor-like protein 2"
82 "CD22 CD22 antigen"
83 "No cluster in current Unigene and no Genbank entry for U77396 (qualifier U77396_at)"
84 "Epb72 gene exon 1"
85 "GB DEF = Fork head domain protein (FKHR) mRNA, 3' end"
86 "PFC Properdin P factor, complement"
87 "Inducible protein mRNA"
88 "PLECKSTRIN"
89 "C-myb gene extracted from Human (c-myb) gene"
90 "IL7R Interleukin 7 receptor"
91 "Cytoplasmic dynein light chain 1 (hdlc1) mRNA"
92 "FOS-RELATED ANTIGEN 2"
