# Supplementary material for: Fast approximate inference for variable selection in Dirichlet process mixtures, with an application to pan-cancer proteomics
Source: Stat Appl Genet Mol Biol. Author manuscript; Available in PMC 2023 Jan 3. (PMC7614016; doi:10.1515/sagmb-2018-0065)
Supplement: Supplementary File [file EMS158447-supplement-Supplementary_File.zip › j_sagmb-2018-0065_suppl/sugs_manuscript_supplement_revised.pdf]

Original Research Article

Oliver M. Crook*, Laurent Gatto, and Paul D. W. Kirk*

Supplementary material for: Fast approximate inference for Dirichlet process mixtures with variable selection: an application to pan-cancer proteomics

SUPPLEMENTAL MATERIALS

DP Gaussian mixture with variable selection

In this supplement, we give specific details for our algorithm in the case of Gaussian mixtures. We note that whenever conjugate priors are chosen all the formulas presented here are available analytically. We specify the mean and covariance matrix associated cluster k by $\theta_k = (m_k, \Sigma_k)$, where $m_k = (m_{k,1}, ..., m_{k,d})$ and Σ_k is diagonal under our independence assumption. The prior on the parameters $p_0(\theta_{k,d})$ is chosen as conjugate normal inverse-chi-squared $(NI\chi^2)$ prior, which is a simple reparameterisation of the normal inverse-gamma prior and is the special case of normal inverse-Wishart prior in one dimension. The specification is as follows:

$$p_0(\theta_{k,d}) \sim N\left(m_{k,d}|\mu_0, \frac{\Sigma_{k,d}}{\lambda_0}\right) I\chi^2\left(\Sigma_{k,d}|\nu_0, S_0\right), \tag{1}$$

with known hyperparameters $\mu_0, \lambda_0, \nu_0, S_0$. Updating the prior (1) with the data from observations $1, ..., (i-1)$ results in

$$p(\theta_{k,d}|x_{-i}, z_{-i}) \sim N\left(m_{k,d}|m^{(i-1)}, \frac{\Sigma_{k,d}}{\lambda^{(i-1)}}\right) I\chi^2\left(\Sigma_{k,d}|\nu^{(i-1)}, S^{(i-1)}\right), \tag{2}$$

***Corresponding author: Oliver M. Crook,** Department of Applied Mathematics and Theoretical Physics, University of Cambridge; Cambridge Centre for Proteomics, Department of Biochemistry, University of Cambridge; MRC Biostatistics Unit, School of Clinical Medicine, University of Cambridge E-mail: omc25@cam.ac.uk
Laurent Gatto, de Duve Institute, UCLouvain, Belgium
***Corresponding author: Paul D. W. Kirk,** MRC Biostatistics Unit, School of Clinical Medicine, University of Cambridge, E-mail: paul.kirk@mrc-bsu.cam.ac.uk

where the parameter updates are obtained sequentially, through the following equations (dropping the subscript d for clarity) (Murphy, 2007):

$$\begin{aligned} m_k^{(i)} &= \frac{\lambda_k^{(i-1)} m_k^{(i-1)} + x_i}{\lambda^{(i)}} \\ \lambda_k^{(i)} &= \lambda_k^{(i-1)} + 1 \\ \nu_k^{(i)} &= \nu_k^{(i-1)} + 1 \\ T_k^{(i)} &= T_k^{(i-1)} + x_i^2 \\ \nu_k^{(i)} S_k^{(i)} &= T_k^{(i)} - \lambda_k^{(i)} \left(m_k^{(i)}\right)^2, \end{aligned} \tag{3}$$

where in the case $i = 0$ the parameters are given by their specified prior values, except we set $T_k^{(0)} = \nu_0 S_0 + \lambda_0 \mu_0^2$. The required conditional likelihood is given by a non-central $\mathcal{T}$-distribution. Remembering at the ith iteration we compute with the updated parameters from the previous iteration; that is, at the $(i-1)^{th}$ iteration, the required distribution is

$$\mathcal{T}\left(\cdot|m_k^{(i-1)}, \nu_k^{(i-1)}, \frac{(1+\lambda_k^{(i-1)})S_k^{(i-1)}}{\lambda_k^{(i-1)}}\right).$$

For a $NI\chi^2$ prior the marginal likelihood is given by the following equation, (dropping the subscript d for clarity)

$$\int_{\theta_k} f(X_k|\theta_k) p_0(\theta_k) d\theta_k = \frac{1}{\pi^{n_k/2}} \frac{\Gamma(\nu_k/2)}{\Gamma(\nu_0/2)} \left(\frac{\lambda_0 (\nu_0 S_0)^{\nu_0}}{\lambda_k (\nu_k S_k)^{\nu_k}}\right)^{1/2}. \tag{4}$$

The other required equations have already been given and require simple substitutions.

Prior Settings for the SUGS and SUGS VarSel high-dimensional example

Here we state the prior specification for the SUGS and SUGS VarSel algorithms. We let μ_0 be the mean of the observations' data for each variable, $\lambda_0 = 0.01$, ν_0 be the number of variables, $S_0 = 0.2$ for all variables. We let $\hat{\beta} = (0.01, 0.1, 1, 5, 10, 15, 30, 50, 100)^T$ and set the prior to be $\mathcal{G}(1,1)$. In addition, for SUGS VarSel we suppose that *a priori* variables are equally likely to be relevant or irrelevant.

Summarising the Bayesian model averaged co-clustering matricies

Fritsch and Ickstadt (2009) propose a method to summarise the posterior similarity matrix of a Bayesian clustering method. We apply their methodology to summarise our Bayesian model averaged co-clustering matrix. They present several method to obtain a clustering by maximising the posterior expected adjusted Rand index. We use the proposed method which obtains clusterings from applying hierarchical clustering with average linkage. An optimal clustering is then obtain by cutting the dendrogram at 0.5 (Fritsch and Ickstadt, 2009).

Gene selection table Genes selected in the SUGSVarSel algorithm applied to the Golub dataset

1 "TCL1 gene (T cell leukemia) extracted from H.sapiens mRNA for Tcell leukemia/lymphoma 1"
2 "TCRB T-cell receptor, beta cluster"
3 "INTERLEUKIN-8 PRECURSOR"
4 "TCRB T-cell receptor, beta cluster"
5 "GB DEF = MAL gene exon 4"
6 "Interleukin 8 (IL8) gene"
7 "GB DEF = (lambda) DNA for immunoglobin light chain"
8 "CST3 Cystatin C (amyloid angiopathy and cerebral hemorrhage)"
9 "CD24 signal transducer mRNA and 3' region"
10 "IGHM Immunoglobulin mu"
11 "MPO Myeloperoxidase"
12 "MHC class II HLA-DP light chain mRNA"
13 "GB DEF = Cystic fibrosis antigen mRNA"
14 "LTB Lymphotoxin-beta"
15 "Major Histocompatibility Complex, Class Ii Beta W52"
16 "CD9 CD9 antigen"
17 "MB-1 gene"
18 "DF D component of complement (adipsin)"
19 "PROBABLE PROTEIN DISULFIDE ISOMERASE ER-60 PRECURSOR"
20 "LGALS3 Lectin, galactoside-binding, soluble, 3 (galectin 3) (NOTE: redefinition of symbol)"
21 "LYZ Lysozyme"
22 "ANX1 Annexin I (lipocortin I)"
23 "IGB Immunoglobulin-associated beta (B29)"
24 "Azurocidin gene"
25 "Na,K-ATPase gamma subunit mRNA"
26 "CD1B CD1b antigen (thymocyte antigen)"
27 "TCF7 Transcription factor 7 (T-cell specific)"
28 "Mac25"
29 "PSAP Sulfated glycoprotein 1"
30 "Terminal transferase mRNA"
31 "CALGRANULIN A"
32 "CLASS II HISTOCOMPATIBILITY ANTIGEN, M ALPHA CHAIN PRECURSOR"
33 "SELL Leukocyte adhesion protein beta subunit"
34 "GB DEF = T-lymphocyte specific protein tyrosine kinase p56lck (lck) abberant mRNA"
35 "MEF2C MADS box transcription enhancer factor 2, polypeptide C (myocyte enhancer factor 2C)"
36 "PRG1 Proteoglycan 1, secretory granule"
37 "CD2 CD2 antigen (p50), sheep red blood cell receptor"
38 "CTGF Connective tissue growth factor"
39 "Lymphoid-restricted membrane protein (Jaw1) mRNA"
40 "GRO2 GRO2 oncogene"
41 "SEF2-1A protein (SEF2-1A) mRNA, 5' end"
42 "GB DEF = T-cell antigen receptor gene T3-delta"
43 "CYSTATIN A"
44 "NPY Neuropeptide Y"
45 "Amphiregulin (AR) gene"
46 "ELA2 Elastatse 2, neutrophil"

47 "SNRPN Small nuclear ribonucleoprotein polypeptide N"
48 "Adenosine triphosphatase, calcium"
49 "MHC cell surface glycoprotein (HLA-DQA) mRNA, 3'end"
50 "Zyxin"
51 "HU-K4 mRNA"
52 "GLUTATHIONE S-TRANSFERASE, MICROSOMAL"
53 "GB DEF = CD1 R2 gene for MHC-related antigen"
54 "GB DEF = Neutrophil elastase gene, exon 5"
55 "LYZ Lysozyme"
56 "Fc-epsilon-receptor gamma-chain mRNA"
57 "HLA-DRB1 Major histocompatibility complex, class II, DR beta 5"
58 "Lysozyme gene (EC 3.2.1.17)"
59 "LYZ Lysozyme"
60 "Pre-B cell enhancing factor (PBEF) mRNA"
61 "LPAP gene"
62 "CHIT1 Chitinase 1"
63 "TCRG T cell receptor gamma chain"
64 "MXS1 Membrane component, X chromosome, surface marker 1"
65 "FLN1 Filamin 1 (actin-binding protein-280)"
66 "BLK Protein-tyrosine kinase blk"
67 "DP2 (Humdp2) mRNA"
68 "HLA CLASS II HISTOCOMPATIBILITY ANTIGEN, DR ALPHA CHAIN PRECURSOR"
69 "Quiescin (Q6) mRNA, partial cds"
70 "GB DEF = Immunoglobulin mu, part of exon 8"
71 "ICAM3 Intercellular adhesion molecule 3"
72 "NF-IL6-beta protein mRNA"
73 "Nuclear Factor Nf-Il6"
74 "Protein-tyrosine-phosphatase (tissue type: foreskin)"
75 "DAGK1 Diacylglycerol kinase, alpha (80kD)"
76 "CD72 CD72 antigen"
77 "GB DEF = Selenoprotein W (selW) mRNA"
78 "PROBABLE G PROTEIN-COUPLED RECEPTOR LCR1 HOMOLOG"
79 "ARHG Ras homolog gene family, member G (rho G)"
80 "CA2 Carbonic anhydrase II"
81 "APLP2 Amyloid beta (A4) precursor-like protein 2"
82 "CD22 CD22 antigen"
83 "No cluster in current Unigene and no Genbank entry for U77396 (qualifier U77396_at)"
84 "Epb72 gene exon 1"
85 "GB DEF = Fork head domain protein (FKHR) mRNA, 3' end"
86 "PFC Properdin P factor, complement"
87 "Inducible protein mRNA"
88 "PLECKSTRIN"
89 "C-myb gene extracted from Human (c-myb) gene"
90 "IL7R Interleukin 7 receptor"
91 "Cytoplasmic dynein light chain 1 (hdlc1) mRNA"
92 "FOS-RELATED ANTIGEN 2"

Pan-cancer proteomics cancer types

The cancers used in the pan-cancer proteomic analysis are Uterine Corpus Endometrial Carcinoma (UCEC), Ovarian (OV), Kidney Renal Clear Cell Carcinoma (KIRC), Colon Adenocarcinoma (COAD), glioblastoma multiforme (GBM), Rectum adenocarcinoma (READ), Thyroid carcinoma (THCA), Kidney renal papillary cell carcinoma (KIRP), Pancreas adenocarcinoma (PAAD), Adenoid cystic carcinoma (ACC), Urothelial Bladder Carcinoma (BLCA), breast cancer (BRCA), Head and neck squamous cell carcinoma (HNSC), Lower grade glioma (LGG), Lung adenocarcinoma (LUAD), Lung Squamous Cell Carcinoma (LUSC), Prostate Adenocarcinoma (PRAD), skin cutaneous melanoma (metastatic and primary) (SKCM) and Stomach Adenocarcinoma (STAD).

Pan-cancer proteomics hierachical clustering

We visualise the data in a heatmap and perform hierarchical clustering on the tumour sample and variables using Euclidean distance, which is usually how such data is analysed (Akbani *et al.*, 2014).

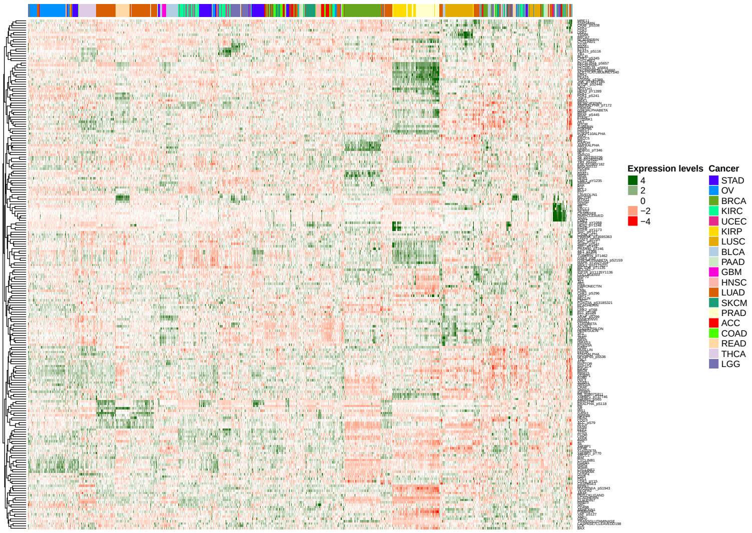

Fig. 0.1: A heatmap the TCPA dataset with hierarchical clustering of tumours and variables. The dendrogram for the tumours is omitted for clarity, but the ordering is retained. The colour bar demonstrates the cancer types.

Cancer clustering table A table of the 18 clusters with more than 20 tumours, produce by the SUGS VarSel algorithm.

	A	B	C	D	E	F	G	H	I	J	K	L	M	N	O	P	Q	R
UCEC	325	43	1	1	11	2	0	0	0	0	0	0	0	0	0	0	0	0
OV	363	42	0	0	0	2	0	0	0	0	0	0	0	0	0	0	0	0
KIRC	3	25	0	2	0	0	405	0	0	0	0	0	0	0	0	0	0	0
COAD	18	33	229	0	28	17	0	0	0	1	0	0	0	0	0	0	0	0
READ	8	13	92	1	10	4	0	0	0	0	0	0	0	0	0	0	0	0
GBM	5	6	0	4	0	0	0	170	2	0	0	0	0	0	0	0	0	0
THCA	0	177	0	3	0	0	0	0	0	0	64	128	0	0	0	0	0	0
KIRP	7	40	0	2	0	0	0	0	0	0	0	0	158	0	0	0	0	0
PAAD	5	72	0	2	3	0	0	0	0	0	0	0	0	23	0	0	0	0
ACC	6	6	0	0	1	0	0	0	0	0	0	0	33	0	0	0	0	0
BLCA	66	46	1	0	3	0	0	0	0	6	0	0	0	0	0	0	0	0
BRCA	151	192	0	34	0	0	0	0	0	1	0	0	0	0	415	0	0	0
HNSC	24	49	0	1	16	3	0	0	0	104	0	0	0	0	0	1	0	0
LGG	0	1	0	0	0	0	0	128	124	0	0	0	0	0	0	0	0	0
LUAD	12	19	0	0	1	11	0	0	0	164	0	0	0	0	0	16	0	0
LUSC	20	5	0	0	10	3	0	0	0	146	0	0	0	0	0	5	0	0
PRAD	3	5	0	1	0	0	0	0	0	0	0	0	0	0	0	0	155	0
SKCM	149	46	0	4	6	0	0	0	0	0	0	0	0	0	0	0	0	0
STAD	8	66	99	2	25	45	0	0	0	0	0	0	0	0	0	0	0	38

References

Akbani, R., Ng, P. K. S., Werner, H. M. J., Shahmoradgoli, M., Zhang, F., Ju, Z., Liu, W., Yang, J.-Y., Yoshihara, K., Li, J., Ling, S., Seviour, E. G., Ram, P. T., Minna, J. D., Diao, L., Tong, P., Heymach, J. V., Hill, S. M., Dondelinger, F., Städler, N., Byers, L. A., Meric-Bernstam, F., Weinstein, J. N., Broom, B. M., Verhaak, R. G. W., Liang, H., Mukherjee, S., Lu, Y., and Mills, G. B. (2014). A pan-cancer proteomic perspective on The Cancer Genome Atlas. *Nature communications*, **5**, 3887.

Fritsch, A. and Ickstadt, K. (2009). Improved criteria for clustering based on the posterior similarity matrix. *Bayesian Anal.*, **4**(2), 367–391.

Murphy, K. P. (2007). Conjugate bayesian analysis of the gaussian distribution. *def*, **1**, 16.
