# Supplementary figures and images for: Fast approximate inference for variable selection in Dirichlet process mixtures, with an application to pan-cancer proteomics
Source: Stat Appl Genet Mol Biol. Author manuscript; Available in PMC 2023 Jan 3. (PMC7614016; doi:10.1515/sagmb-2018-0065)

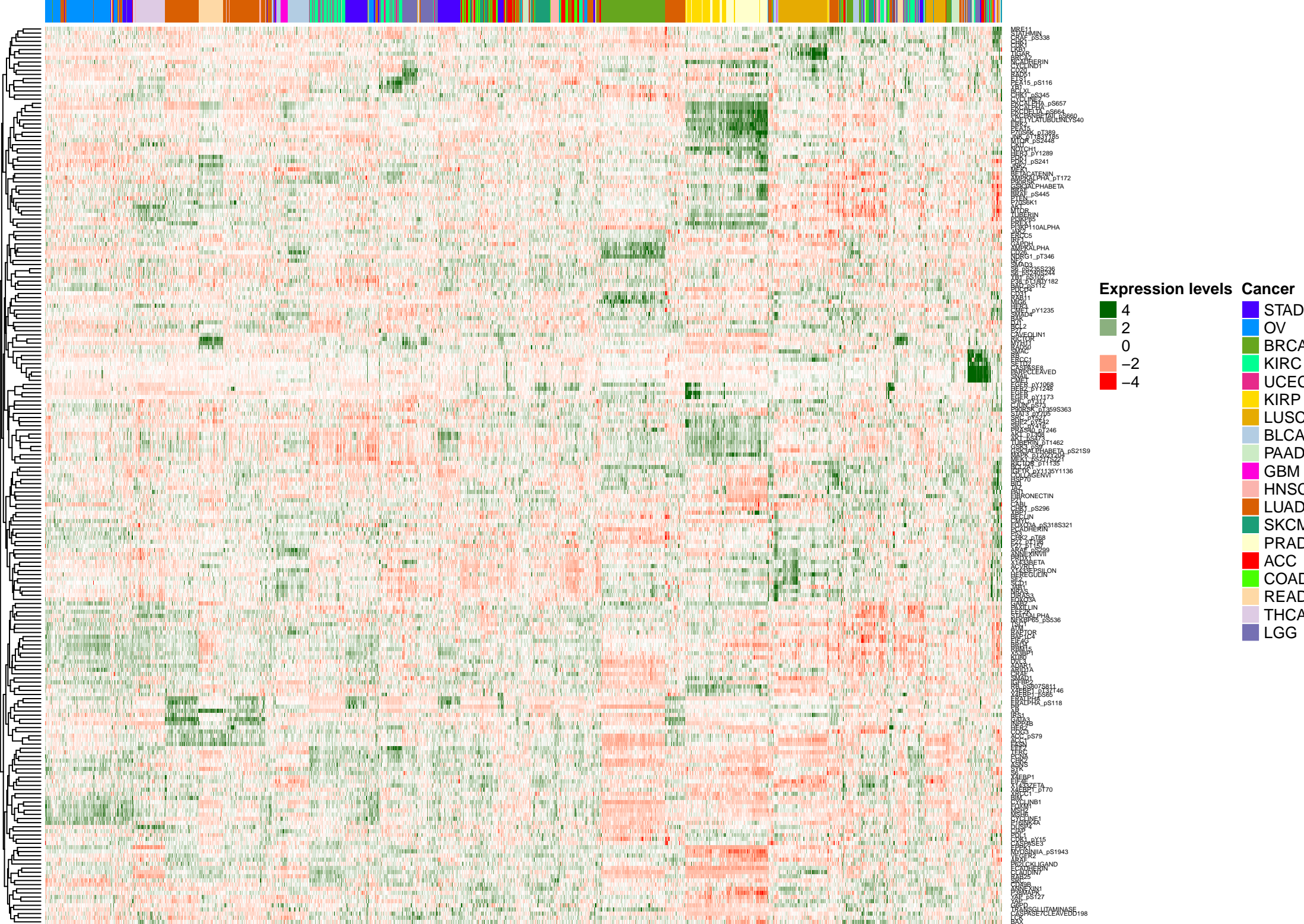

Supplement: Supplementary File [file EMS158447-supplement-Supplementary_File.zip › j_sagmb-2018-0065_suppl/TCPAheathclust.pdf]
